# CKD and CKDu in northern Peru: a cross-sectional analysis under the DEGREE protocol

**DOI:** 10.1186/s12882-021-02239-8

**Published:** 2021-01-21

**Authors:** Andrea Ruiz-Alejos, Ben Caplin, J. Jaime Miranda, Neil Pearce, Antonio Bernabé-Ortiz

**Affiliations:** 1 CRONICAS Center of Excellence in Chronic Diseases, Universidad Peruana Cayetano Heredia, Av. Armendariz 445, Miraflores, Lima, Peru; 2 Department of Medicine, School of Medicine, Universidad Peruana Cayetano Heredia, Lima, Peru; 3 Centre for Nephrology, University College London, London, UK; 4 Department of Medical Statistics and Non-communicable Disease Epidemiology, London School of Hygiene and Tropical Medicine, London, UK; 5 Centre for Global Health, London School of Hygiene and Tropical Medicine, London, UK

**Keywords:** Chronic kidney disease, CKDu, Impaired kidney function, Hypertension, Obstructive nephropathy

## Abstract

**Background:**

This study estimated the prevalence and risk factors for decreased estimated glomerular filtration rate (eGFR) in those without known hypertension, type 2 diabetes mellitus, or heavy proteinuria as a surrogate marker for chronic kidney disease of unknown cause (CKDu) among adults in the North of Peru.

**Methods:**

A cross-sectional study was conducted following the Disadvantaged Populations eGFR Epidemiology (DEGREE) Study protocol. Low eGFR was defined based on a single eGFR ≤60 mL/min/1.7m^2^ estimated using the CKD-EPI equation. Environmental conditions related to CKDu (i.e., work in agriculture or sugarcane, water source, heat intolerance, and pesticide exposure) were evaluated, in addition to traditional risk factors for CKD (i.e., smoking, heavy drinking, physical activity, hypertension, type 2 diabetes mellitus, urolithiasis, among others).

**Results:**

A total of 1514 subjects were included in the study, mean age 45.1 (SD: 16.4), and 55.2% were females. Overall, only 26 cases (1.7%; 95%CI: 1.1–2.5%) had an eGFR < 60 mL/min/1.7m^2^ compatible with CKD definition; when those with hypertension and type-2 diabetes or heavy proteinuria were excluded, according to the DEGREE protocol, the estimate fell to 0.9% (95%CI: 0.4–1.5%). Low physical activity levels (OR = 1.99; 95%CI: 1.18–3.34), hypertension (OR = 2.07; 1.26–3.41), and urolithiasis (OR = 1.97; 95%CI: 1.18–3.27) were factors associated with low eGFR.

**Conclusions:**

A low population-based prevalence of low eGFR (as a surrogate for CKDu), both in rural and urban settings areas, in the Northern Peru, was found. Low physical activity levels, hypertension and urolithiasis were factors associated with low eGFR. Interventions to prevent CKD cases may be focused on well-known CV risk factors and urolithiasis.

**Supplementary Information:**

The online version contains supplementary material available at 10.1186/s12882-021-02239-8.

## Background

In less than a decade, chronic kidney disease (CKD) has increased from the 18th to the 6th leading cause of death worldwide, affecting nearly 850 million people and causing 2.4 million deaths annually [1]. Age-related non-communicable diseases (NCDs) and conditions such as hypertension (HTN) and type 2 diabetes mellitus (T2DM) are the main risk factors associated with CKD; in addition, other causes include autoimmune disease, congenital abnormalities of the urinary tract, and obstructive uropathy. Recently, cases of chronic kidney disease of unknown cause (CKDu), a condition not related to NCDs, age, or any of the above aetiologies, have emerged as a significant problem in South Asia and Central America [2, 3].

CKDu is also referred to by various regional names including Meso-American Nephropathy (Pacific Coast of Central America) [4], Sri Lankan Nephropathy [5], and Uddanam Nephropathy [6], affecting mostly young farmers between 30 and 50 years old. The causes of CKDu are unknown [7], but the hypothesized causes include pesticide exposure and heavy metal poisoning associated with agriculture work, as well as heat stress in low-altitude areas. The latter hypothesis proposes that environmental heat, in combination with recurrent dehydration and high-levels of physical exertion, lead to recurrent acute kidney injury episodes and eventually CKD [8]. However, CKDu has not been reported in other countries where this combination of risk factors could be found [9, 10].

Latin America, mainly Mexico, has amongst the highest rates globally of renal replacement therapy (RRT), including haemodialysis, peritoneal dialysis, and kidney transplant [11]. However, epidemiologic data on CKD are mostly from Brazil, and a few other countries in South America. Although, in the Brazilian northeast region the temperature could regularly rise over 38 °C (100 °F), neither a higher prevalence of CKD, nor CKDu cases, have been reported among Brazilian agricultural workers [12]. Peru, a country closer to the Equator, has reported a CKD prevalence of 13 to 21% [13, 14], which is comparable to that in El Salvador and Nicaragua [15, 16]. Approximately 50% of the end stage population of CKD in Peru does not receive dialysis or renal replacement therapy (RRT), since the health system does not have the necessary resources (technological and human) [17, 18]. Moreover, in Peru, 2.0% of the deaths at the national level were attributable to CKD, being in the top ten causes of death [19].

Therefore, the purpose of this study was to estimate the prevalence of low eGFR (< 60 ml/min /1.7 m^2^) in those without known HTN, T2DM or heavy proteinuria as a surrogate marker for CKDu among participants from the Tumbes region, in the North of Peru. In addition, we assessed the factors associated with the prevalence of impaired kidney function (i.e., low eGFR) in general and surrogate CKDu in particular.

## Methods

### Study design and setting

A cross-sectional study was conducted following the protocol of the Disadvantaged Populations eGFR Epidemiology Study (DEGREE) [20]. This study was conducted in Tumbes, a region located in the North of Peru, on the border with Ecuador, and where approximately 90% of the population is urban. In addition, nearly 20 and 1% of the population respectively are considered poor and extremely poor, and 15% do not have basic sanitation services [21].

Tumbes was chosen for the following reasons (see Table 1 for comparison with other regions where CKDu has been documented): agriculture workers (up to 10% of the active economic population), contamination of the Rio Puyango, the main source of water (i.e., cadmium and arsenic), and warm climate with low precipitation rates. The temperature of the city has an annual average temperature of 27 °C, reaching almost 35 °C during the summer [22].

**Table 1:** Environmental and demographic characteristics of Tumbes and regions with CKDu

	Tumbes	Andhra Pradesh/Uddanam	El Salvador (coastal region)	Egypt El-Sharkia	Sri Lanka	Nicaragua
Population	240,590	14,807	238,244	8,017,894	21,358,975	206,264
Urbanicity	94.1% (urban)	24% (urban)	67% (urban)	77% (urban)	77% (urban)	81.6% (urban)
Climate	Arid and subtropical	Hot tropical	Tropical	Arid	Tropical	Tropical
Humidity	76%	70–90%	62–100%	Over 90%	70–95%	65–80%
Precipitation	131.4 mm	1067 mm	13 to 155 mm	4 mm (no rain season)	200 mm	181 mm
Temperature	21–40 °C	34.3 °C	23–35 °C	32.6 °C	36 °C	35 °C
Average annual temperature	25.3 °C	31.5 °C	26 °C	26.2 °C	27 °C	32.5 °C
Altitude	Sea level	60–70 m	90 m	4–8 m	81 m	109 m
Agriculture	Rice, lime, banana	Rice coconuts and cashew	Sugarcane	Cotton	Chena, rice cultivations	Banana, sugarcane
Pesticide	Paraquat, methomyl 90SP	Organochlorides	Paraquat	Cadmium, nickel and mercury	Glyphosate	Pyrethroids and chlorpyrifos
Metal contamination	Arsenic	Silica	Arsenic, cadmium	Cadmium	Cadmium	Aluminum, arsenic, lead
Leptospira	0.02–5%	61.8%	–	49.7%	53%	36%
Hypertension rates	26.9%	38.5–615%	30%	–	26%	22%
Type 2 diabetes mellitus	10.3%	7.2–30%	8.8%	–	8.6%	10%
CKDu	< 1%	1.6–4.8%, up to 60%	25%	17.7%	15–21%	19%

### Study participants

Adults aged ≥18 years, resident in Tumbes, were invited to participate using the most updated census in the area (2014). Pregnant women, those who declined to give blood and urine samples, and those with mobility disabilities preventing bioimpedance assessment, were excluded from the study. The sample was stratified by population group, so that, participants were from urban and rural areas in similar proportions (50%). In addition, we decided not to recruit more women after their number reached 60% for each of these two subgroups.

### Outcome

We calculated the estimated Glomerular Filtration Rate (eGFR) using the Chronic Kidney Disease – Epidemiology Collaboration (CKD-EPI) equation. We defined three categories of kidney function based on eGFR results: moderate or established kidney dysfunction (< 60 mL/min/1.7m^2^), mild kidney dysfunction (60–90 mL/min/1.7m^2^), and normal (≥90 mL/min/1.7m^2^). CKD was defined as the proportion of individuals with moderate or established kidney function (eGFR < 60 mL/min/1.7m^2^) in the overall sample; whereas the prevalence of CKDu was estimated using the same indicator but excluding individuals with previous diagnosis of HT and/or T2DM, and those having a heavy proteinuria, defined as 3+ in urine dipstick (equivalent to ≥ 300 mg/dl of protein) according to the DEGREE protocol [20].

### Questionnaires

During the first visit, questionnaires were used to gather information on socio-demographic factors (age, sex) and socioeconomic status (education, employment status, house-hold income and health insurance). Environmental conditions associated with CKDu (previous or current work in agriculture or sugarcane, water source, heat, and pesticide exposure) were addressed. Information on past medical history was focused on cardiovascular diseases (HT, T2DM, myocardial infarction, stroke, and hypercholesterolemia), and behavioural risk factors (alcohol, smoking, and physical activity). We also included questions on CKD and its associated causes, including congenital kidney malformation, diabetic nephropathy, polycystic kidney disease, urolithiasis, use of nephrotoxic medications (non-steroidal anti-inflammatory, parenteral use of antibiotics, and herbal medicine), tuberculosis, hepatitis B, and leptospirosis.

### Clinical assessment

Participants were invited for another visit to obtain blood samples for fasting glucose and creatinine, urine samples (dipstick urinalysis) and clinical examination. We asked the participants to refrain from eating meat, caffeine, tobacco and paracetamol 8 h prior to taking the sample. The clinical evaluation included three blood pressure measurements taken after 5 min of resting (seated) position and 5 min apart from each other (evaluated with an automatic digital calibrated sphygmomanometer OMRON HEM-780, Tokyo, Japan), stand height (cm), weight (kg) by standardized procedures, and body composition (i.e. body fat percentage), for which we used a supine bioimpedance analyzer balance (BodyStat 1500, BodyStat Limited, British Islands) calibrated at a single frequency (50 Hz).

### Analysis of biological samples

Urine dipstick analyses yielded semi-quantitative values ​​of blood, proteins, leukocytes, glucose, pH and urine density. We measured serum creatinine in 5 ml of blood sample using a mass spectrometry method calibrated according to isotope dilution (IDMS, English acronym). As in the DEGREE Study protocol, serum samples were stored at − 20 °C for future Cystatin C determination.

### Other variables of interest

We also gathered information on key reported risk factors for CKD and CKDu. Age was considered as both a continuous and categorical variable. Level of education was classified in three categories (< 7, 7–11, ≥12 years of education). We also considered socioeconomic status as monthly household income dichotomised with a cut-off point of 850 PEN (250 USD, minimum salary wage at the moment of the study).

We also collected information on ever working in agriculture, sugarcane, and pesticide use; these variables were built as categorical (yes/no). Due to potential sources of contamination of water, we chose *access to piped water* (yes/no) as the variable to be assessed. Heat intolerance, a proxy of heat exposure, was considered to have occurred if the participant reported to have fainted when exposed to hot weather. We built a variable analgesic/antibiotic use due to the variety of potential nephrotoxic medications (including herbal medicine use) usually sold without prescriptions. For this, we focused on the ever use of analgesics and intramuscular antibiotics. In addition, history of some diseases because their impact on CKD was also included: tuberculosis, hepatitis B, and leptospirosis.

Heavy drinking (consuming at least 6 alcoholic standard beverages, monthly), and current smoking within the last 12 months were also included. Physical activity was dichotomised in low vs. moderate/high according to the International Physical Activity Questionnaire (IPAQ). Body mass index (BMI) was calculated according to the WHO recommendations, and then split into three categories (< 25 kg/m^2^, between 25 and < 30 kg/m^2^, and ≥30 kg/m^2^). HT was defined as a systolic blood pressure ≥ 140 mmHg or diastolic blood pressure ≥ 90 mmHg and/or previous diagnosis by a physician and current medication. To estimate the values for systolic and diastolic blood pressure, we used the mean of the last two (out of three) measurements conducted. T2DM was defined as a fasting (8–12 h) glucose ≥126 mg/dl or self-report of previous diagnosis and treatment. The cut-off point for glycosuria was ≥250 mg/dl, urinary density ≥1020 (as a proxy for dehydration) and proteinuria if the urine level marker of protein was ≥30 mg/dl (equivalent to 1+ in urine dipstick).

### Sample size

We planned a sample size of 750 participants in each population group (rural and urban). Assuming an expected CKDu prevalence of 5%, with 1500 individuals in total, we will have a precision of 1%. In addition, this precision would be of 1.6% if only 750 subjects are evaluated as in each study group.

### Data analysis

We conducted descriptive analyses of socio-demographic, socioeconomic, cardiovascular risk profile and CKD risk profiles according to the study population group (rural and urban) and kidney function. These comparisons were conducted for the overall sample (compatible with CKD) and among those without known HT, T2DM or heavy proteinuria (as a proxy of CKDu). Chi-square tests for comparison of categorical variables, and Student’s t test or Mann-Whitney U was used for comparison of numerical variables. In addition, the prevalence of CKD and CKDu were also estimated with their respective 95% confidence intervals (95%CI).

As very few cases with eGFR levels compatible with CKD and CKDu (i.e., eGFR < 60 mL/min/1.7m^2^) were found, we analysed factors associated with impaired kidney function (eGFR < 90 mL/min/1.7m^2^) rather than factors associated with eGFR< 60. We used logistic regression models to determine associated factors for impaired kidney function. As a result, crude model and adjusted models are shown. The first adjusted regression model was controlled by age (as continuous variable) and sex; whereas the second regression model included also population group and education level as potential confounders.

## Results

### Characteristics of the study participants

A total of 1514 of 1818 (83.3%) contacted subjects consented to be included in the study. Socio-demographic, occupational and lifestyle characteristics of the overall sample are summarized in Supplementary Table 1. More than half of participants were < 50 years old (overall mean age 45.1 [SD: 16.4] years) and 55.2% were females. The employment rate was 55.9% and higher in men (89.4%) than women (28.6%). Only 33.9% of the study population reported to have worked in agriculture and were mostly male rural dwellers (56.2%). The prevalence of measured HT was 15.6% (mean SBP: 115.7 [SD: 18.9]) and increased to 20.8% when self-reported HT diagnosis was added (Supplementary Table 2). The mean fasting glucose was 95.9 (SD: 39.5) mg/dl, with a prevalence of 8.3% for T2DM. The mean BMI was 27.5 (SD: 4.5) kg/m^2^ with a prevalence of 43.5% for overweight and 26.8% for obesity. A total of 274 (18.1%) participants self-reported a previous diagnosis of CKD, although mean creatinine was 0.7 (SD: 0.2) mg/dl, and urolithiasis was reported in 15.7% of the total sample (Supplementary Table 3).

After excluding those with known HT, T2DM or heavy proteinuria, a total of 1272 subjects were analysed. Socio-demographic and lifestyle characteristics of this population are summarized in Table 2. Over 70% of the subjects were younger than 50 years and over half of them were employed, 20.8% reported having worked with pesticides, and 33.7% had worked in agriculture. Almost all the urban dwellers had access to piped water. Undiagnosed HT and T2DM were found in 11.2 and 2.1% of the population (Table 3). Of note, only 2 (0.2%) had proteinuria (1+ in urine dipstick).

**Table 2:** Sociodemographic, occupational and lifestyle characteristics by study group and sex (*n* = 1272)

	Urban		Rural	
	Male	Female	Male	Female
	**(*n*** **= 261)**	**(*n*** **= 364)**	**(*n*** **= 298)**	**(*n*** **= 349)**
Age, mean (SD)	45.2 (16.6)	41.9 (14.4)	45.0 (15.9)	39.3 (14.4)
Age (categorized)				
< 50 years	165 (63.2)	266 (73.1)	191 (64.1)	274 (78.5)
50+ years	96 (36.8)	98 (26.9)	107 (35.9)	75 (21.5)
Education (in years)				
0–7 years	69 (26.4)	96 (26.4)	115 (38.6)	129 (36.9)
7–11 years	128 (49.1)	161 (44.2)	125 (42.0)	136 (39.0)
> 12 years	64 (24.5)	107 (29.4)	58 (19.5)	84 (24.1)
Work				
Employed	229 (87.8)	112 (30.7)	286 (96.0)	99 (28.4)
Student	10 (3.8)	18 (5.0)	6 (2.0)	15 (4.3)
Homemaker & unpaid	0 (0.0)	223 (61.3)	1 (0.3)	223 (63.8)
retired	10 (3.8)	0 (0.0)	0 (0.0)	2 (0.6)
Unemployed	11 (4.6)	11 (3.0)	5 (1.7)	10 (2.9)
Pesticide exposure (ever)				
Yes	28 (10.7)	5 (1.4)	199 (66.8)	32 (9.2)
Sugarcane work				
Yes	3 (1.2)	1 (0.3)	18 (6.0)	6 (1.7)
Agriculture work				
Yes	53 (20.3)	9 (2.5)	259 (86.9)	107 (30.7)
Heat exposure at work				
Yes	97 (37.2)	16 (4.4)	187 (62.8)	71 (20.3)
Monthly household income				
< 850 PEN (≈258 USD)	110 (48.9)	194 (64.9)	159 (61.2)	231 (78.6)
Water source				
Piped water	232 (99.1)	305 (100.0)	10 (3.4)	15 (4.4)
Well	2 (0.9)	0 (0.0)	193 (66.4)	229 (67.2)
River	0 (0.0)	0 (0.0)	62 (21.3)	54 (15.8)
Trunk	0 (0.0)	0 (0.0)	26 (8.9)	43 (12.6)
Health insurance				
Yes	224 (85.8)	330 (90.7)	239 (80.6)	315 (90.3)
Current smoking				
Yes	63 (24.1)	5 (1.4)	81 (27.2)	6 (1.7)
Heavy drinking				
Yes	53 (20.3)	7 (1.9)	67 (22.5)	3 (0.9)
Physical activity				
Low	189 (72.4)	334 (91.8)	164 (55.0)	288 (82.5)
Medicine intake risk				
Yes	100 (38.3)	222 (61.0)	129 (43.3)	194 (55.6)
Herbal medicine use				
Yes	108 (41.4)	154 (42.3)	158 (53.0)	181 (51.9)

**Table 3:** Clinical characteristics by study group and sex (n = 1272)

	Urban		Rural	
	Male	Female	Male	Female
	(***n =*** 261)	(***n =*** 364)	(***n =*** 298)	(***n =*** 349)
SBP, mean (SD)	118.3 (13.8)	107.7 (13.5)	120.0 (16.6)	106.9 (14.9)
DBP, mean (SD)	79.5 (9.9)	75.4 (9.4)	78.8 (10.6)	73.8 (9.9)
Hypertension				
Yes	37 (14.2)	23 (6.3)	54 (18.1)	29 (8.3)
Previous stroke				
Yes	0 (0.0)	1 (0.3)	0 (0.0)	2 (0.6)
High cholesterol				
Yes	29 (11.1)	59 (16.3)	31 (10.7)	89 (26.3)
Tuberculosis				
Yes	5 (1.9)	2 (0.6)	9 (3.0)	5 (1.4)
Hepatitis B				
Yes	0 (0.0)	3 (0.8)	6 (1.4)	1 (0.3)
Leptospirosis				
Yes	0 (0.0)	2 (0.6)	2 (0.7)	1 (0.3)
CKD (self-report)				
Yes	50 (19.2)	67 (18.4)	42 (14.1)	55 (16.1)
Urolithiasis				
Yes	46 (17.6)	58 (15.9)	38 (12.8)	50 (14.3)
Glucose, mean (SD)	88.6 (16.9)	90.3 (20.2)	88.7 (16.0)	88.8 (21.7)
Type 2 diabetes				
Yes	4 (1.5)	9 (2.5)	5 (1.7)	9 (2.6)
Body fat %, mean (SD)	23.6 (6.0)	38.4 (6.3)	23.5 (5.7)	38.3 (6.3)
Body mass index, mean (SD)	27.3 (4.4)	28.5 (4.6)	25.7 (3.8)	27.5 (4.5)
BMI categorized				
Normal	77 (29.5)	83 (22.8)	129 (43.3)	102 (29.2)
Overweight	122 (46.7)	152 (41.8)	128 (43.0)	151 (43.3)
Obese	62 (23.8)	129 (35.4)	41 (13.7)	96 (27.5)
Creatinine, mean (SD)	0.9 (0.2)	0.6 (0.1)	0.9 (0.1)	0.6 (0.1)
eGFR, mean (SD)	100.6 (17.4)	111.5 (16.0)	104.1 (16.0)	114.0 (17.3)
Urine density				
≥1020	166 (63.6)	203 (55.8)	216 (72.5)	230 (65.9)
Urine protein				
Negative	260 (99.6)	363 (99.7)	297 (99.7)	348 (99.7)
Trace	0 (0.0)	1 (0.3)	1 (0.3)	0 (0.0)
Positive	1 (0.4)	0 (0.0)	0 (0.0)	1 (0.3)
Glucosuria				
≥ 250 mg/dl	6 (2.3)	7 (1.9)	1 (0.3)	5 (1.4)

### Prevalence of low eGFR and proxy CKDu

In the overall sample, and according to the CKD-EPI formula, only 26 cases (1.7%; 95%CI: 1.1–2.5%) had an eGFR < 60 mL/min/1.7m^2^ compatible with the definition of CKD, whereas 16.6% (95%CI: 14.8–18.6%) had and eGFR ≥60 but < 90 mL/min/1.7m^2^. When these estimates were calculated in the subsample according to the DEGREE protocol, only 11 individuals (0.9%; 95%CI: 0.4–1.5%) had an eGFR < 60 mL/min/1.7m^2^, compatible with CKDu.

In bivariate analysis, those with impaired kidney function (eGFR < 60 mL/min/1.7m^2^) were older, male, with low education level, reported having worked in agriculture, had more access to piped water, reported using herbal medicine, had higher systolic and diastolic blood pressure, and consequently higher HTN prevalence, and low urine density (Table 4).

**Table 4:** Baseline characteristics of study population by eGFR results (*n =* 1272)

	eGFR categories			***p***-value
	< 60	60–90	≥90	
	**(*n*** **= 11)**	**(*n*** **= 165)**	**(*n*** **= 1096)**	
Age, mean (SD)	66.5 (13.8)	62.4 (14.4)	39.3 (12.9)	< 0.001
Age categorized				
< 50 years	1 (9.1)	34 (20.6)	861 (78.6)	< 0.001
50+ years	10 (90.9)	129 (79.4)	235 (21.4)	
Sex				
Male	8 (72.7)	107 (64.9)	444 (40.5)	< 0.001
Female	3 (27.3)	58 (35.1)	652 (59.5)	
Site				
Urban	7 (63.6)	95 (57.6)	523 (47.7)	0.04
Rural	4 (36.7)	70 (42.4)	573 (52.3)	
Education (in years)				
0–7 years	8 (72.7)	110 (66.7)	291 (26.6)	< 0.001
7–11 years	3 (27.3)	38 (23.0)	509 (46.4)	
> 12 years	0 (0.0)	17 (10.3)	296 (27.0)	
Pesticide exposure (ever)				
Yes	2 (18.1)	35 (21.2)	227 (20.7)	0.97
Sugarcane work				
Yes	1 (9.1)	3 (1.8)	24 (2.2)	0.28
Agriculture work				
Yes	4 (36.4)	69 (41.8)	355 (32.4)	0.06
Heat exposure at work				
Yes	3 (27.3)	52 (31.5)	316 (28.8)	0.77
Monthly household income				
< 850 PEN (≈258 USD)	3 (50.0)	97 (69.3)	594 (63.7)	0.34
Water source				
Piped water	6 (60.0)	90 (58.4)	465 (46.2)	0.007
Well	2 (20.0)	39 (25.3)	383 (38.1)	
River	0 (0.0)	19 (12.4)	97 (9.6)	
Trunk	2 (20.0)	6 (3.9)	61 (6.1)	
Health insurance				
Yes	11 (100.0)	150 (90.9)	947 (86.4)	0.12
Current smoking				
Yes	1 (9.1)	20 (12.1)	134 (12.2)	0.95
Heavy drinking				
Yes	0 (0.0)	12 (7.3)	118 (10.8)	0.21
Physical activity				
Low	8 (72.7)	137 (83.0)	830 (75.7)	0.11
Medicine intake risk				
Yes	4 (36.4)	82 (49.7)	559 (51.0)	0.60
Herbal medicine use				
Yes	7 (63.6)	98 (59.4)	496 (45.3)	0.002
SBP, mean (SD)	130.8 (26.1)	125.4 (19.7)	110.4 (14.0)	< 0.001
DBP, mean (SD)	83.5 (14.8)	81.1 (11.5)	75.8 (9.8)	< 0.001
Hypertension				
Yes	4 (36.4)	44 (26.7)	95 (8.7)	< 0.001
High cholesterol				
Yes	2 (18.2)	36 (22.0)	170 (15.8)	0.14
Tuberculosis				
Yes	0 (0.0)	3 (1.8)	18 (1.7)	0.90
Hepatitis B				
Yes	0 (0.0)	1 (0.6)	7 (0.6)	0.97
Leptospirosis				
Yes	0 (0.0)	0 (0.0)	5 (0.5)	0.67
Urolithiasis				
Yes	2 (18.2)	33 (20.0)	157 (14.3)	0.16
Glucose, mean (SD)	104.4 (54.6)	91.7 (19.1)	88.7 (18.4)	0.005
Type 2 diabetes				
Yes	1 (9.1)	4 (2.4)	22 (2.0)	0.26
Body fat %, mean (SD)	32.8 (10.2)	33.6 (9.5)	31.7 (9.5)	0.05
Body mass index, mean (SD)	25.9 (4.5)	27.2 (4.3)	27.4 (4.5)	0.54
BMI categorized				
Normal	5 (45.5)	52 (31.5)	334 (30.5)	0.64
Overweight	5 (45.5)	74 (44.9)	474 (43.3)	
Obese	1 (9.0)	39 (23.6)	288 (26.2)	
Urine density				
≥1020	4 (36.4)	78 (47.3)	733 (66.9)	< 0.001
Urine protein				
Negative	10 (90.9)	165 (100.0)	1093 (99.7)	< 0.001
Trace	1 (9.1)	0	1 (0.1)	
Positive	0 (0.0)	0	2 (0.2)	
Urine glucose				
≥ 250 mg/dl	1 (9.1)	0 (0.0)	18 (1.6)	0.03

### Factors associated with impaired kidney function (eGFR < 90 mL/min/1.7m^2^)

Table 5 shows the findings adjusted for age and sex (model 1) and further adjusted for population group and education. In the fully adjusted model, low physical activity levels (OR = 1.99; 95% CI: 1.18–3.34), HTN (OR = 2.07; 95% CI: 1.26–3.41), and urolithiasis (OR = 1.97; 95% CI: 1.18–3.27) were factors associated with impaired kidney function. On the other hand, sugarcane work was associated with lower odds of impaired kidney function (OR = 0.28; 95% CI: 0.08–0.96) in adjusted model. The same factors, but not sugarcane work, were associated with impaired kidney function when the analysis was conducted with the entire study sample (Supplementary Table 4).

**Table 5:** Factors associated with impaired kidney function*: adjusted models (*n =* 1272)

	Adjusted model 1	Adjusted model 2
	OR (95%CI)	OR (95%CI)
Pesticide exposure (yes)	**0.55 (0.33–0.92)**	0.70 (0.39–1.25)
Sugarcane work (yes)	**0.25 (0.07–0.82)**	**0.28 (0.08–0.96)**
Agriculture work (yes)	**0.55 (0.35–0.86)**	0.72 (0.40–1.29)
Heat exposure at work (yes)	0.72 (0.45–1.14)	0.84 (0.51–1.37)
Water source (access to piped water)	**1.66 (1.11–2.49)**	0.96 (0.42–2.20)
Current smoking (yes)	0.66 (0.36–1.23)	0.66 (0.36–1.23)
Heavy drinking (yes)	0.91 (0.44–1.87)	0.97 (0.47–2.01)
Physical activity (low levels)	**2.23 (1.24–3.71)**	**1.99 (1.18–3.34)**
Medicine intake risk (yes)	1.03 (0.69–1.54)	1.04 (0.69–1.56)
Herbal medicine use (yes)	0.99 (0.66–1.48)	1.05 (0.69–1.58)
Hypertension (yes)	**1.94 (1.19–3.17)**	**2.07 (1.26–3.41)**
Type 2 diabetes (yes)	0.99 (0.31–3.13)	1.11 (0.34–3.61)
Body mass index		
Overweight	1.22 (0.76–1.98)	1.17 (0.72–1.91)
Obesity	1.28 (0.74–2.22)	1.11 (0.63–1.96)
Urolithiasis (yes)	**2.06 (1.24–3.40)**	**1.97 (1.18–3.27)**
Tuberculosis (yes)	1.13 (0.28–4.58)	1.20 (0.30–4.81)
Hepatitis B (yes)	1.66 (0.16–16.9)	1.74 (0.16–19.1)
Leptospirosis (yes)	–	–

Bold estimates are statistically significant (*p*<0.05). Impaired kidney function was defined as eGFR < 90 mL/min/1.7m^2^

Adjusted model 1: controlled by age and sex

Adjusted mode 2: controlled by age, sex, population group, and education

## Discussion

### Main findings

The findings of this study are interesting because this has been conducted in a setting with high frequency of environmental exposures that have been hypothesized to be associated with CKDu in other populations. Thus, one might have expected to find a substantial prevalence of low eGFR (surrogate of CKDu), but in fact we found a prevalence of less than 1%, consistent with what has been observed in other ‘non-epidemic’ settings. Factors associated with increased probability of impaired kidney function were HTN, urolithiasis, and low physical activity levels; nevertheless, sugarcane work was associated with low probability of impaired kidney function but only in the DEGREE protocol subsample and not in the entire sample.

### Comparison with previous studies

There is strong evidence that CKDu exists in Central America [23] and Sri Lanka [24]. There have been unconfirmed case reports in other locations such as India [25], Saudi Arabia [26], and Egypt and Senegal [27]. A cross-sectional study found CKDu prevalence in most affected areas of Sri Lanka was as high as 22.9% [24]. Globally, the increasing prevalence of end-stage CKD in African American, Hispanic and Native American populations is at least 50% attributable to T2DM [20]. In contrast, the contribution of CKDu to overall CKD burden in Peru seems to be negligible.

Tumbes is a region located on the border with Ecuador, and the inhabitants have a constant exposure to a hot and sub-tropical weather. Most of the regions where CKDu has been reported have a tropical environment, with humidity levels above 85%; however, Tumbes climate is arid, and thus, subtropical, and humidity tends to be lower, compared to Uddanam, El Salvador and Nicaragua. Nevertheless, considering the variability of tropical climate, the annual average ranges of humidity, precipitation, and annual temperature in the hotspots for CKDu are not considerably different (Table 1).

Despite the fact that exposures to agrochemicals and heavy metal are frequent in Tumbes region [28], we could not find a high number of cases of CKDu. Mining activities in Ecuador led to heavy metal contamination of Rio Puyango [29], the main source of water for dwellers in Tumbes; and nearly 60 and 90% of the rural male dwellers from our study reported previous pesticide exposure and having worked in agriculture, respectively. The evidence supporting pesticide use and its relationship with CKDu relies on chronic exposure more than acute major nephrotoxic damage [30]. Thus, male farmers from Chichigalpa (Nicaragua) had one of the highest prevalence of impaired kidney function in the region (41.9%) and this was reported to be associated with sugarcane cutting and pesticide inhalation [31]. A relatively recent systematic review found no association between CKDu and pesticide exposure [32], which was the case in our study. Surprisingly, sugarcane work was associated with lower odds of impaired kidney function, perhaps due to residual confounding or over-adjustment as few individuals reported work in sugarcane.

The factors that we found to be associated with impaired kidney function in our study are consistent with those widely reported in literature, and are not related to CKDu. For example, a recent systematic review has reported that combined aerobic and resistance exercise improves renal function, especially among patients with CKD [33]. HTN and obstructive nephropathy have been described as main causes of impaired kidney function.

### Public health relevance

CKD has emerged as a public health concern in Peru due to its increasing mortality and lack of early detection. A study using healthcare and death records from the Peruvian Ministry of Health evidenced an increase of 300% of CKD prevalence between 2010 and 2016, especially in the Tumbes region [34]. In addition, another study reported that the proportionate mortality due to CKD has also increased in this region during the last 15 years [19].

Despite of this, cases of CKD are mainly attributed to HTN and T2DM, but also obstructive nephropathy and low physical activity levels have been described. This study shows that very few cases are probably due to CKDu, even though the region selected for this study may have several environmental and weather conditions that have been hypothesized to cause CKDu; these include humidity, temperature, pesticide use, and metal contamination (Table 1). From the public health perspective, interventions to prevent CKD cases may be focused on well-known CV risk factors (i.e. HTN and T2DM) as well as promoting physical activity and prevent or appropriately treat urolithiasis. According to our results, it does not appear that CKDu is a major problem, at least in the Tumbes region. This finding is potentially important internationally, since the cause(s) of CKDu are still unknown, but the main hypotheses involve heat and pesticide exposure – we have found CKDu to not be present in a region which has these hypothesized causal exposures.

### Strengths and limitations

This exploratory study has several strengths including appropriate sample size, high response rates, representativeness, and internationally referenced laboratory validation. The sample size was considered to analyse associated factors of CKD and CKDu among rural and urban dwellers, as performed in previous studies [35, 36]. As expected, recruited subjects showed a similar risk profile for CKD and CKDu considering environmental and socio-demographic factors.

Limitations, however, are also present. First, this is a cross-sectional study, and we are not able to confirm that the relevant exposures (e.g. pesticides) occurred before the relevant outcomes (low eGFR). Nevertheless, the focus of the study was to determine the prevalence of CKD and CKDu and factors evaluated were those which are considered as potential causes of these conditions. Second, only a single eGFR measurement was carried out, so we were not able to confirm cases of CKD as we were unable to demonstrate chronicity – however, this would not affect our main finding that CKDu prevalence was very low. In addition, the CKD–EPI equation has not been validated in Peru and may potentially underestimate the number of cases with eGFR< 60, especially among women [37]. Nonetheless, due to the low prevalence of eGFR< 60 and even < 90 in young adults, it is unlikely that this would explain our findings. In addition to that, an eGFR< 90 alone has not been associated with the prediction of poor outcomes (e.g. cardiovascular disease, end-stage kidney disease, etc). Third, recall bias may be a concern as we collected information by using self-report of current and past behaviours and other exposures. In addition, some questions (i.e. heat intolerance or analgesic/antibiotic use) used to capture information were not previously validated. Finally, selection bias may arise as only one Peruvian region with the environmental characteristics for CKDu was chosen for the study.

## Conclusions

Despite having appropriate environmental conditions and work-related stressors that have been hypothesized to cause CKDu, this study found a low population-based prevalence of CKDu, both in rural and urban settings areas in Tumbes, a region in the Northern Peru. Low physical activity levels, HTN and urolithiasis were the factors associated with CKD in this sample. From the public health perspective, interventions to prevent or delay CKD cases may be focused on well-known CV risk factors and urolithiasis.

## Supplementary Information

**Additional file 1: Supplementary Table 1:** Sociodemographic, occupational and lifestyle characteristics by study group and sex (*n* = 1514). **Supplementary Table 2:** Clinical characteristics and chronic kidney risk factors by study group and sex (n = 1514). **Supplementary Table 3:** Baseline characteristics of study population by eGFR results (*n =* 1514). **Supplementary Table 4:** Factors associated with kidney impaired function: adjusted models (*n =* 1514).

## Data Availability

The dataset used and/or analyzed during the current study are available from the corresponding author on reasonable request.

## Abbreviations

CKD: Chronic kidney disease
CKDu: Chronic kidney disease of unknown cause
CKD-EPI: Chronic Kidney Disease – Epidemiology Collaboration
CV: Cardiovascular
DEGREE: Disadvantaged Populations eGFR Epidemiology Study
eGFR: Estimated glomerular filtration rate
IPAQ: International Physical Activity Questionnaire
NCD: Non-communicable diseases
HTN: Hypertension
T2DM: Type 2 diabetes mellitus
WHO: World Health Organization

## References

[1] Crews DC, Bello AK, Saadi G (2019). Burden, access, and disparities in kidney disease. Saudi J Kidney Dis Transplant.

[2] Caplin B, Yang CW, Anand S, Levin A, Madero M, Saran R, Jayasinghe S, De Broe M, Yeates K, Tonelli M (2019). The International Society of Nephrology's international consortium of collaborators on chronic kidney disease of unknown etiology: report of the working group on approaches to population-level detection strategies and recommendations for a minimum dataset. Kidney Int.

[3] Jha V, Garcia-Garcia G, Iseki K, Li Z, Naicker S, Plattner B, Saran R, Wang AY, Yang CW (2013). Chronic kidney disease: global dimension and perspectives. Lancet (London, England).

[4] Wesseling C, Crowe J, Hogstedt C, Jakobsson K, Lucas R, Wegman D (2012). Mesoamerican Nephropathy: Report from the First International Research Workshop on MeN.

[5] Athuraliya NT, Abeysekera TD, Amerasinghe PH, Kumarasiri R, Bandara P, Karunaratne U, Milton AH, Jones AL (2011). Uncertain etiologies of proteinuric-chronic kidney disease in rural Sri Lanka. Kidney Int.

[6] Ganguli A (2016). Uddanam nephropathy/regional nephropathy in India: preliminary findings and a Plea for further research. Am J Kidney Dis.

[7] Pearce N, Caplin B (2019). Let's take the heat out of the CKDu debate: more evidence is needed. Occup Environ Med.

[8] Johnson RJ, Wesseling C, Newman LS (2019). Chronic kidney disease of unknown cause in agricultural communities. N Engl J Med.

[9] Anand S, Shivashankar R, Ali MK, Kondal D, Binukumar B, Montez-Rath ME, Ajay VS, Pradeepa R, Deepa M, Gupta R (2015). Prevalence of chronic kidney disease in two major Indian cities and projections for associated cardiovascular disease. Kidney Int.

[10] Stanifer JW, Jing B, Tolan S, Helmke N, Mukerjee R, Naicker S, Patel U (2014). The epidemiology of chronic kidney disease in sub-Saharan Africa: a systematic review and meta-analysis. Lancet Glob Health.

[11] Francis ER, Allen AK, Herrera-Anazco P, Kuo CC, Cardenas MK, Feldman HI, Baral SD, Miranda JJ (2016). Establishing a higher priority for chronic kidney disease in Peru. Lancet Glob Health.

[12] Paula Santos U, Zanetta DM, Terra-Filho M, Burdmann EA (2015). Burnt sugarcane harvesting is associated with acute renal dysfunction. Kidney Int.

[13] Francis ER, Kuo CC, Bernabe-Ortiz A, Nessel L, Gilman RH, Checkley W, Miranda JJ, Feldman HI (2015). Burden of chronic kidney disease in resource-limited settings from Peru: a population-based study. BMC Nephrol.

[14] Herrera-Anazco P, Taype-Rondan A, Lazo-Porras M, Alberto Quintanilla E, Ortiz-Soriano VM, Hernandez AV (2017). Prevalence of chronic kidney disease in Peruvian primary care setting. BMC Nephrol.

[15] Ferguson R, Leatherman S, Fiore M, Minnings K, Mosco M, Kaufman J, Kerns E, Amador JJ, Brooks DR, Fiore M (2020). Prevalence and risk factors for CKD in the general population of southwestern Nicaragua. J Am Soc Nephrol.

[16] Lunyera J, Mohottige D, Von Isenburg M, Jeuland M, Patel UD, Stanifer JW (2016). CKD of uncertain etiology: a systematic review. Clin J Am Soc Nephrol.

[17] Herrera-Anazco P, Benites-Zapata VA, Leon-Yurivilca I, Huarcaya-Cotaquispe R, Silveira-Chau M (2015). Chronic kidney disease in Peru: a challenge for a country with an emerging economy. J Bras Nefrol.

[18] Zevallos L, Pastor R, Moscoso B (2011). Supply and demand of medical specialists in the health facilities of the Ministry of Health: national, regional and by type of specialty gaps. Rev Peru Med Exp Salud Publica.

[19] Carrillo-Larco RM, Bernabe-Ortiz A (2018). Mortality from chronic kidney disease in Peru: national trends 2003-2015. Rev Peru Med Exp Salud Publica.

[20] Caplin B, Jakobsson K, Glaser J, Nitsch D, Jha V, Singh A, Correa-Rotter R, Pearce N (2017). International collaboration for the epidemiology of eGFR in low and middle income populations - rationale and core protocol for the disadvantaged populations eGFR epidemiology study (DEGREE). BMC Nephrol.

[21] Nacionales C (2017). XII de Población, VII de Vivienda y III de Comunidades Indígenas.

[22] Instituto Nacional de Estadistica e Informatica (2015). Peru: Temperatura promedio, maxima y minima, segun departamento, 2015.

[23] Correa-Rotter R, Wesseling C, Johnson RJ (2014). CKD of unknown origin in Central America: the case for a Mesoamerican nephropathy. Am J Kidney Dis.

[24] Jayatilake N, Mendis S, Maheepala P, Mehta FR (2013). Chronic kidney disease of uncertain aetiology: prevalence and causative factors in a developing country. BMC Nephrol.

[25] Singh AK, Farag YM, Mittal BV, Subramanian KK, Reddy SR, Acharya VN, Almeida AF, Channakeshavamurthy A, Ballal HS, P G (2013). Epidemiology and risk factors of chronic kidney disease in India - results from the SEEK (screening and early evaluation of kidney disease) study. BMC Nephrol.

[26] El Minshawy O, Ghabrah T, El Bassuoni E (2014). End-stage renal disease in Tabuk area, Saudi Arabia: an epidemiological study. Saudi J Kidney Dis Transplant.

[27] Barsoum RS (2013). Burden of chronic kidney disease: North Africa. Kidney Int Suppl.

[28] Fernandez Yarleque J (2019). Nivel de contaminación por metales pesados: hg, Pb, as y Cianuro (CN-), en el naciente río Binacional Puyango –Tumbes (Perú – Ecuador).

[29] Mora A, Jumbo-Flores D, Gonzalez-Merizalde M, Bermeo-Flores S (2016). Levels of heavy metals in riverine sediments of the Puyango river basin, Ecuador. Rev Int Contam Ambient.

[30] Valcke M, Levasseur ME, Soares da Silva A, Wesseling C (2017). Pesticide exposures and chronic kidney disease of unknown etiology: an epidemiologic review. Environ Health.

[31] Raines N, González M, Wyatt C, Kurzrok M, Pool C, Lemma T, Weiss I, Marín C, Prado V, Marcas E (2014). Risk factors for reduced glomerular filtration rate in a Nicaraguan community affected by Mesoamerican nephropathy. MEDICC Rev.

[32] González-Quiroz M, Pearce N, Caplin B, Nitsch D (2018). What do epidemiological studies tell us about chronic kidney disease of undetermined cause in Meso-America? A systematic review and meta-analysis. Clin Kidney J.

[33] Wu X, Yang L (2020). Effects of combined aerobic and resistance exercise on renal function in adult patients with chronic kidney disease: a systematic review and meta-analysis.

[34] Atamari-Anahui N, Ccorahua-Rios MS, Condori-Huaraka M, Huamanvilca-Yepez Y, Amaya E, Herrera-Añazco P (2020). Epidemiology of chronic kidney disease in Peru and its relation to social determinants of health. Int Health.

[35] Lebov JF, Valladares E, Peña R, Peña EM, Sanoff SL, Cisneros EC, Colindres RE, Morgan DR, Hogan SL (2015). A population-based study of prevalence and risk factors of chronic kidney disease in León, Nicaragua. Can J Kidney Health Dis.

[36] O'Callaghan-Gordo C, Shivashankar R, Anand S, Ghosh S, Glaser J, Gupta R, Jakobsson K, Kondal D, Krishnan A, Mohan S (2019). Prevalence of and risk factors for chronic kidney disease of unknown aetiology in India: secondary data analysis of three population-based cross-sectional studies. BMJ Open.

[37] Weerakkody RM, Sheriff MHR (2019). Predictive performance of the estimating equations of renal function in Sri Lankan subjects. BMC Res Notes.

