# Supplementary material for: CKD and CKDu in northern Peru: a cross-sectional analysis under the DEGREE protocol
Source: BMC Nephrol. 2021 Jan 21;22:37. doi: 10.1186/s12882-021-02239-8 (PMC7818732; doi:10.1186/s12882-021-02239-8)
Supplement: Supplementary file 1 — Additional file 1: Supplementary Table 1: Sociodemographic, occupational and lifestyle characteristics by study group and sex (n = 1514). Supplementary Table 2: Clinical characteristics and chronic kidney risk factors by study group and sex (n = 1514). Supplementary Table 3: Baseline characteristics of study population by eGFR results (n = 1514). Supplementary Table 4: Factors associated with kidney impaired function: adjusted models (n = 1514). [file 12882_2021_2239_MOESM1_ESM.docx]

**Supplementary Table 1:** Sociodemographic, occupational and lifestyle characteristics by study group and sex (n = 1514)

|  | **Urban** | | **Rural** | |
| --- | --- | --- | --- | --- |
|  | **Male** | **Female** | **Male** | **Female** |
|  | **(n = 430)** | **(n = 420)** | **(n = 339)** | **(n = 415)** |
| Age, mean (SD) | 48.3 (17.4) | 44.0 (15.4) | 46.4 (16.4) | 42.4 (16.2) |
| Age (categorized) |  |  |  |  |
| < 50 years | 187 (55.0) | 283 (67.4) | 204 (60.2) | 289 (69.6) |
| 50+ years | 153 (45.0) | 137 (32.6) | 135 (39.8) | 126 (30.4) |
| Education (in years) |  |  |  |  |
| 0-7 years | 109 (32.1) | 130 (30.9) | 143 (42.2) | 174 (41.9) |
| 7 – 11 years | 160 (47.1) | 180 (42.9) | 133 (39.2) | 151 (36.4) |
| >12 years | 71 (20.8) | 110 (26.2) | 63 (18.6) | 90 (21.7) |
| Work |  |  |  |  |
| Employed | 285 (83.8) | 120 (28.6) | 322 (95.0) | 119 (28.7) |
| Student | 10 (2.9) | 18 (4.3) | 6 (1.8) | 15 (3.6) |
| Homemaker & unpaid | 0 (0.0) | 269 (64.0) | 1 (0.3) | 266 (64.1) |
| Retired | 21 (6.2) | 1 (0.2) | 1 (0.3) | 2 (0.5) |
| Unemployed | 24 (7.1) | 12 (2.9) | 9 (2.6) | 13 (3.1) |
| Pesticide exposure (ever) |  |  |  |  |
| Yes | 45 (13.2) | 5 (1.2) | 226 (66.7) | 37 (8.9) |
| Sugarcane work |  |  |  |  |
| Yes | 4 (1.2) | 1 (0.2) | 19 (5.6) | 8 (1.9) |
| Agriculture work |  |  |  |  |
| Yes | 79 (23.2) | 10 (2.4) | 293 (86.4) | 131 (31.6) |
| Heat exposure (work) |  |  |  |  |
| Yes | 121 (35.6) | 16 (3.8) | 213 (62.8) | 81 (19.5) |
| Monthly household income |  |  |  |  |
| <850 PEN (≈258 USD) | 150 (51.7) | 234 (68.4) | 187 (62.3) | 271 (78.3) |
| Water source |  |  |  |  |
| Piped water | 298 (99.0) | 352 (100) | 12 (3.6) | 22 (5.4) |
| Well | 3 (1.0) | 0 (0.0) | 216 (65.3) | 267 (65.6) |
| River | 0 (0.0) | 0 (0.0) | 71 (21.5) | 69 (17.0) |
| Trunk | 0 (0.0) | 0 (0.0) | 32 (9.7) | 49 (12.0) |
| Health insurance |  |  |  |  |
| Yes | 299 (87.9) | 381 (90.7) | 277 (81.7) | 380 (91.6) |
| Current smoking |  |  |  |  |
| Yes | 76 (28.8) | 5 (3.9) | 86 (34.0) | 7 (7.8) |
| Heavy drinking |  |  |  |  |
| Yes | 61 (17.9) | 7 (1.7) | 74 (21.8) | 3 (0.7) |
| Physical activity |  |  |  |  |
| Low | 252 (74.1) | 387 (92.1) | 196 (57.8) | 349 (84.1) |
| Medicine intake risk |  |  |  |  |
| Yes | 134 (39.4) | 262 (62.4) | 149 (44.0) | 236 (56.9) |
| Herbal medicine use |  |  |  |  |
| Yes | 149 (43.8) | 188 (44.8) | 178 (52.5) | 223 (53.7) |

**Supplementary Table 2:** Clinical characteristics and chronic kidney risk factors by study group and sex (n = 1514)

|  | **Urban** | | **Rural** | |
| --- | --- | --- | --- | --- |
|  | **Male** | **Female** | **Male** | **Female** |
|  | **(n = 430)** | **(n = 420)** | **(n = 339)** | **(n = 415)** |
| SBP, mean (SD) | 122.9 (18.2) | 110.0 (15.9) | 122.0 (18.3) | 110.5 (19.2) |
| DBP, mean (SD) | 81.6 (11.6) | 76.3 (10.0) | 79.7 (11.0) | 75.5 (11.8) |
| Hypertension |  |  |  |  |
| Yes | 102 (30.0) | 60 (14.3) | 81 (23.9) | 72 (17.4) |
| Previous stroke |  |  |  |  |
| Yes | 0 (0.0) | 2 (0.5) | 3 (0.9) | 3 (0.7) |
| High cholesterol |  |  |  |  |
| Yes | 59 (17.4) | 84 (20.1) | 48 (14.5) | 122 (30.2) |
| Tuberculosis |  |  |  |  |
| Yes | 6 (1.8) | 2 (0.5) | 11 (3.3) | 8 (1.9) |
| Hepatitis B |  |  |  |  |
| Yes | 2 (0.6) | 4 (1.0) | 6 (1.8) | 2 (0.5) |
| Leptospirosis |  |  |  |  |
| Yes | 0 (0.0) | 2 (0.5) | 3 (0.9) | 1 (0.2) |
| CKD (self-report) |  |  |  |  |
| Yes | 68 (20.0) | 78 (18.6) | 53 (15.6) | 75 (18.1) |
| Urolithiasis |  |  |  |  |
| Yes | 60 (17.7) | 67 (16.0) | 46 (13.6) | 65 (15.7) |
| Glucose, mean (SD) | 95.1 (36.0) | 97.3 (41.3) | 94.1 (34.6) | 96.7 (44.1) |
| Type 2 diabetes |  |  |  |  |
| Yes | 26 (7.7) | 36 (8.6) | 24 (7.1) | 39 (9.4) |
| Body fat %, mean (SD) | 24.5 (6.2) | 39.0 (6.4) | 23.9 (5.7) | 39.0 (6.4) |
| Body mass index, mean (SD) | 27.4 (4.4) | 28.6 (4.6) | 25.8 (3.8) | 27.7 (4.7) |
| BMI categorized |  |  |  |  |
| Normal | 95 (27.9) | 89 (21.2) | 145 (42.8) | 121 (29.2) |
| Overweight | 158 (46.5) | 177 (42.1) | 147 (43.3) | 177 (42.6) |
| Obese | 87 (25.6) | 154 (36.7) | 47 (13.9) | 117 (28.2) |
| Creatinine, mean (SD) | 0.9 (0.2) | 0.6 (0.2) | 0.9 (0.3) | 0.6 (0.1) |
| eGFR, mean (SD) | 97.8 (18.6) | 109.2 (18.2) | 102.4 (18.1) | 111.1 (19.0) |
| Urine density |  |  |  |  |
| ≥1020 | 215 (63.2) | 232 (55.2) | 239 (70.5) | 268 (64.6) |
| Urine protein |  |  |  |  |
| Negative | 336 (98.8) | 418 (99.6) | 337 (99.4) | 414 (99.8) |
| Trace | 0 (0.0) | 1 (0.2) | 1 (0.3) | 0.0 |
| Positive | 4 (1.2) | 1 (0.2) | 1 (0.3) | 1 (0.2) |
| Glucosuria |  |  |  |  |
| ≥ 250 mg/dl | 20 (5.9) | 24 (5.7) | 10 (3.0) | 20 (4.8) |

**Supplementary Table 3:** Baseline characteristics of study population by eGFR results (n = 1514)

|  | **eGFR categories** | | |  |
| --- | --- | --- | --- | --- |
|  | **< 60** | **60-90** | **≥90** | **p-value** |
|  | **(n = 1236)** | **(n = 252)** | **(n = 26)** |  |
| Age, mean (SD) | 69.4 (12.2) | 64.3 (14.1) | 40.6 (13.4) | < 0.001 |
| Age categorized |  |  |  |  |
| < 50 years | 1 (3.8) | 44 (17.5) | 918 (74.3) | <0.001 |
| 50+ years | 25 (96.2) | 208 (82.5) | 318 (25.7) |  |
| Sex |  |  |  |  |
| Male | 15 (57.7) | 161 (63.9) | 503 (40.7) | <0.001 |
| Female | 11 (42.3) | 91 (36.1) | 733 (59.3) |  |
| Site |  |  |  |  |
| Urban | 15 (57.7) | 148 (58.7) | 597 (48.3) | 0.008 |
| Rural | 11 (42.3) | 104 (41.3) | 639 (51.7) |  |
| Education (in years) |  |  |  |  |
| 0-7 years | 19 (73.1) | 173 (68.7) | 364 (29.4) | < 0.001 |
| 7 – 11 years | 5 (19.2) | 57(22.6) | 562 (45.5) |  |
| >12 years | 2 (7.7) | 22 (8.7) | 310 (25.1) |  |
| Pesticide exposure (ever) |  |  |  |  |
| Yes | 5 (19.2) | 59 (23.4) | 249 (20.2) | 0.50 |
| Sugarcane work |  |  |  |  |
| Yes | 1 (3.9) | 5 (2.0) | 26 (2.1) | 0.82 |
| Agriculture work |  |  |  |  |
| Yes | 11 (42.3) | 103 (40.9) | 399 (32.3) | 0.02 |
| Heat exposure (work) |  |  |  |  |
| Yes | 5 (19.2) | 78 (31.0) | 348 (28.2) | 0.38 |
| Monthly household income |  |  |  |  |
| <850 PEN (≈258 USD) | 13 (65.0) | 150 (71.4) | 679 (64.8) | 0.18 |
| Water source |  |  |  |  |
| Piped water | 13 (54.2) | 139 (59.4) | 532 (47.0) | <0.001 |
| Well | 5 (20.8) | 54 (23.1) | 427 (37.7) |  |
| River | 2 (8.3) | 29 (12.4) | 109 (9.6) |  |
| Trunk | 4 (16.7) | 12 (5.1) | 65 (5.7) |  |
| Health insurance |  |  |  |  |
| Yes | 25 (96.2) | 236 (93.7) | 1076 (87.1) | 0.006 |
| Current smoking |  |  |  |  |
| Yes | 1 (5.6) | 27 (17.9) | 146 (25.8) | 0.02 |
| Heavy drinking |  |  |  |  |
| Yes | 0 (0.0) | 19 (7.5) | 126 (10.2) | 0.11 |
| Physical activity |  |  |  |  |
| Low | 22 (84.6) | 212 (84.1) | 950 (76.9) | 0.03 |
| Medicine intake risk |  |  |  |  |
| Yes | 14 (53.9) | 129 (51.2) | 638 (51.6) | 0.99 |
| Herbal medicine use |  |  |  |  |
| Yes | 15 (57.7) | 151 (59.9) | 572 (46.3) | <0.001 |
| SBP, mean (SD) | 142.7 (26.4) | 130.7 (22.6) | 112.1 (15.6) | <0.001 |
| DBP, mean (SD) | 89.2 (14.2) | 83.2 (12.9) | 76.7 (10.5) | <0.001 |
| Hypertension |  |  |  |  |
| Yes | 17 (65.4) | 120 (47.6) | 178 (14.4) | <0.001 |
| High cholesterol |  |  |  |  |
| Yes | 7 (26.9) | 77 (30.7) | 229 (18.8) | < 0.001 |
| Tuberculosis |  |  |  |  |
| Yes | 1 (3.9) | 6 (2.4) | 20 (1.6) | 0.52 |
| Hepatitis B |  |  |  |  |
| Yes | 1 (3.9) | 3 (1.2) | 10 (0.8) | 0.25 |
| Leptospirosis |  |  |  |  |
| Yes | 0 (0.0) | 1 (0.4) | 5 (0.4) | 0.95 |
| Urolithiasis |  |  |  |  |
| Yes | 4 (15.4) | 54 (21.4) | 180 (14.6) | 0.02 |
| Glucose, mean (SD) | 122.4 (84.6) | 96.1 (28.6) | 95.3 (39.8) | 0.003 |
| Type 2 diabetes |  |  |  |  |
| Yes | 7 (26.9) | 29 (11.5) | 89 (7.2) | <0.001 |
| Body fat %, mean (SD) | 35.0 (9.8) | 34.1 (9.6) | 32.0 (9.6) | 0.004 |
| Body mass index, mean (SD) | 26.9 (3.9) | 27.3 (4.4) | 27.5 (4.6) | 0.69 |
| BMI categorized |  |  |  |  |
| Normal | 9 (34.6) | 73 (29.0) | 368 (29.7) | 0.66 |
| Overweight | 13 (50.0) | 115 (45.6) | 531 (43.0) |  |
| Obese | 4 (15.4) | 64 (25.4) | 337 (27.3) |  |
| Urine density |  |  |  |  |
| ≥1020 | 10 (38.5) | 119 (47.2) | 825 (66.8) | <0.001 |
| Urine protein |  |  |  |  |
| Negative | 22 (84.6) | 252 (100) | 1231 (99.6) | <0.001 |
| Trace | 1 (3.9) | 0 | 1 (0.1) |  |
| Positive | 3 (11.5) | 0 | 4 (0.3) |  |
| Urine glucose |  |  |  |  |
| ≥ 250 mg/dl | 4 (15.4) | 9 (3.6) | 61 (4.9) | 0.03 |

**Supplementary Table 4:** Factors associated with kidney impaired function*: adjusted models (n = 1514)

|  | **Adjusted model 1** | **Adjusted model 2** |
| --- | --- | --- |
|  | **OR (95%CI)** | **OR (95%CI)** |
| Pesticide exposure (yes) | 0.70 (0.45 – 1.08) | 0.90 (0.56 – 1.46) |
| Sugarcane work (yes) | **0.31 (0.11 – 0.85)** | 0.35 (0.13 – 1.01) |
| Agriculture work (yes) | **0.66 (0.45 – 0.96)** | 0.90 (0.56 – 1.44) |
| Heat exposure at work (yes) | 0.85 (0.57 – 1.28) | 1.01 (0.67 – 1.54) |
| Water source (access to piped water) | **1.48 (1.05 – 2.07)** | 0.87 (0.46 – 1.64) |
| Current smoking (yes) | 0.68 (0.39 – 1.18) | 0.68 (0.39- 1.18) |
| Heavy drinking (yes) | 1.19 (0.65 – 2.18) | 1.26 (0.68 – 2.34) |
| Physical activity (low levels) | **1.96 (1.26 – 3.03)** | **1.79 (1.15 – 2.80)** |
| Medicine intake risk (yes) | 1.16 (0.82 – 1.64) | 1.16 (0.82 – 1.63) |
| Herbal medicine use (yes) | 1.12 (0.79 – 1.57) | 1.19 (0.84 – 1.68) |
| Hypertension (yes) | **2.32 (1.62 – 3.31)** | **2.33 (1. 62 – 3.33)** |
| Type 2 diabetes (yes) | 0.76 (0.46 – 1.27) | 0.78 (0.47 – 1.30) |
| Body mass index |  |  |
| Overweight | 1.35 (0.89 – 2.05) | 1.27 (0.84 – 1.94) |
| Obesity | 1.27 (0.80 – 2.04) | 1.11 (0.68 – 1.79) |
| Urolithiasis (yes) | **2.04 (1.34 – 3.11)** | **1.98 (1.30 – 3.02)** |
| Tuberculosis (yes) | 1.58 (0.51 – 4.84) | 1.76 (0.58 – 5.31) |
| Hepatitis B (yes) | 2.31 (0.52 – 10.2) | 2.35 (0.51 – 10.9) |
| Leptospirosis (yes) | 1.44 (0.12 – 17.4) | 1.77 (0.16 – 20.3) |

Impaired kidney function was defined as eGFR <<90 mL/min/1.7m^2^

Adjusted model 1: controlled by age and sex

Adjusted mode 2: controlled by age, sex, population group, and education.
